# Prevalence of Rare Diseases in Consanguineous Marriages: A Case of Ullrich Muscular Dystrophy in a Resource‐Limited Setting

**DOI:** 10.1002/ccr3.73658

**Published:** 2026-10-11

**Authors:** Kanza Farhan, Muhammad Burhan Tariq, Amna Farhan, Erma Khalid, Suhaira Hafeez Syed, Mohammed Hammad Jaber Amin

**Affiliations:** ^1^ Sindh Medical College Jinnah Sindh Medical University Karachi Pakistan; ^2^ Shaheed Mohtarma Benazir Bhutto Medical College, Lyari Karachi Pakistan; ^3^ National Institute of Child Health/National Institute of Cardiovascular Diseases Karachi Pakistan; ^4^ Jinnah Postgraduate Medical Center Karachi Pakistan; ^5^ Faculty of Medicine Alzaiem Alazhari University Khartoum Sudan

**Keywords:** case report, consanguinity, genetic myopathy, neonatal hypotonia, Pakistan, rare disease, respiratory distress, tertiary care, Ullrich congenital muscular dystrophy

## Abstract

In regions with high consanguinity, rare recessive disorders like Ullrich congenital muscular dystrophy (UCMD) may present at birth with hypotonia and respiratory distress. To enhance quality of life and prevent recurrence in future pregnancies, early recognition, genetic counseling, and care from a multidisciplinary team of specialists are essential.

## Learning Points

- Ullrich congenital muscular dystrophy (UCMD) should be evaluated in newborns presenting with hypotonia, respiratory distress, and musculoskeletal abnormalities, particularly within consanguineous families.
- In resource‐limited settings, diagnoses typically need more time due to insufficient genetic testing and expert treatment. Although solutions are unavailable, early physiotherapy, orthopedic supervision, and respiratory assistance might enhance quality of life.
- Genetic counseling and familial screening are crucial to prevent recurrence in future pregnancies. Low‐ and middle‐income countries (LMICs) require immediate establishment of national registries and infrastructure for rare illnesses.

## Graphical abstract

Ullrich Muscular Dystrophy in Consanguineous Marriage.

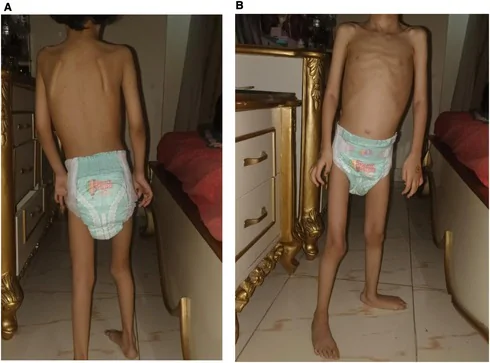

## 1 Introduction

Ullrich congenital muscular dystrophy (UCMD) is an uncommon neuromuscular condition that manifests in early childhood and belongs to the category of collagen VI‐related myopathies. It is related to mutations in the COL6A1, COL6A2, or COL6A3 genes [1, 2, 3]. These genes encode essential components of the extracellular matrix that maintain the integrity of muscle fibers. UCMD is characterized by a triad of issues: proximal muscle weakness, distal joint stiffness, and hypermobile joints. These issues are commonly observed in infants [3]. Individuals impacted typically exhibit delayed motor milestones, exacerbating respiratory issues, and ultimately lose the capacity to ambulate in early adolescence [4]. Kyphoscoliosis, soft dermis, and autonomously developing keloids are other potential signs. UCMD can be inherited through either an autosomal dominant or recessive pattern. The recessive variant is typically associated with a more severe clinical prognosis [5].

Information regarding the global prevalence of UCMD is limited, while estimates indicate it occurs in fewer than 1 in 1,000,000 live births. The true impact is likely not fully comprehended, particularly in regions where access to genetic testing, neuromuscular knowledge, and public health infrastructure is limited [6]. The majority of known occurrences globally remain infrequent, and there are few cases recorded in countries with numerous genetic risk factors.

Consanguinity is a significant genetic risk factor for rare autosomal recessive disorders. Approximately 65% of marriages in Pakistan occur between individuals who are related, with over 50% of these unions involving first cousins. This significantly increases the likelihood of homozygosity for deleterious genes, thereby elevating the incidence of hereditary disorders such as UCMD [7]. Despite the elevated risk, individuals remain mostly uninformed about or undiagnosed with uncommon neuromuscular illnesses, particularly in public healthcare environments with limited resources. Identifying and treating rare diseases at an early stage is more challenging due to the absence of national registries, genetic counseling programs, and affordable testing methods.

This case report discusses an infant male with UCMD, born to first cousins, who received treatment at a tertiary care facility in Pakistan with limited diagnostic capabilities. The diagnosis was determined using a clinical phenotypic assessment and a neurophysiological assessment. This case is noteworthy not just due to the rarity of the condition but also because it occurred within a familial household. This illustrates the interplay between genetic inheritance and public health issues in developing nations. It highlights the pressing necessity for improved genetic services, early diagnostic methods, and community‐level awareness to address the concealed burden of uncommon congenital disorders in Pakistan.

## 2 Case Presentation

### 2.1 Patient Information

The patient is a female infant born at 37 weeks gestation to a 27‐year‐old mother experiencing her first pregnancy, which was complicated by uncontrolled gestational hypertension. The parents are first cousins residing in an urban area of Pakistan. The family exhibited no recorded history of neuromuscular disorders. The patient is the firstborn, and both the index child and her younger sibling exhibit evidence of hypotonia, suggesting a potential inherited neuromuscular disorder.

### 2.2 Clinical Findings

Upon birth, the infant exhibited flaccidity, hypotonia, and cyanosis, accompanied by severe respiratory distress. Apgar scores were undocumented. She demonstrated global muscle weakness with 0/5 strength in all four limbs, bilateral talipes valgus, a hyperextended wrist posture, facial ptosis, and probable scoliosis (Figure 1A,B). The physical examination indicated left‐sided developmental dysplasia of the hip (DDH) and widespread ligamentous laxity. No indications of cranial nerve involvement were present, and there were no dysmorphic facial characteristics save for ptosis. The neurological evaluation was generally unremarkable.

**FIGURE 1:**
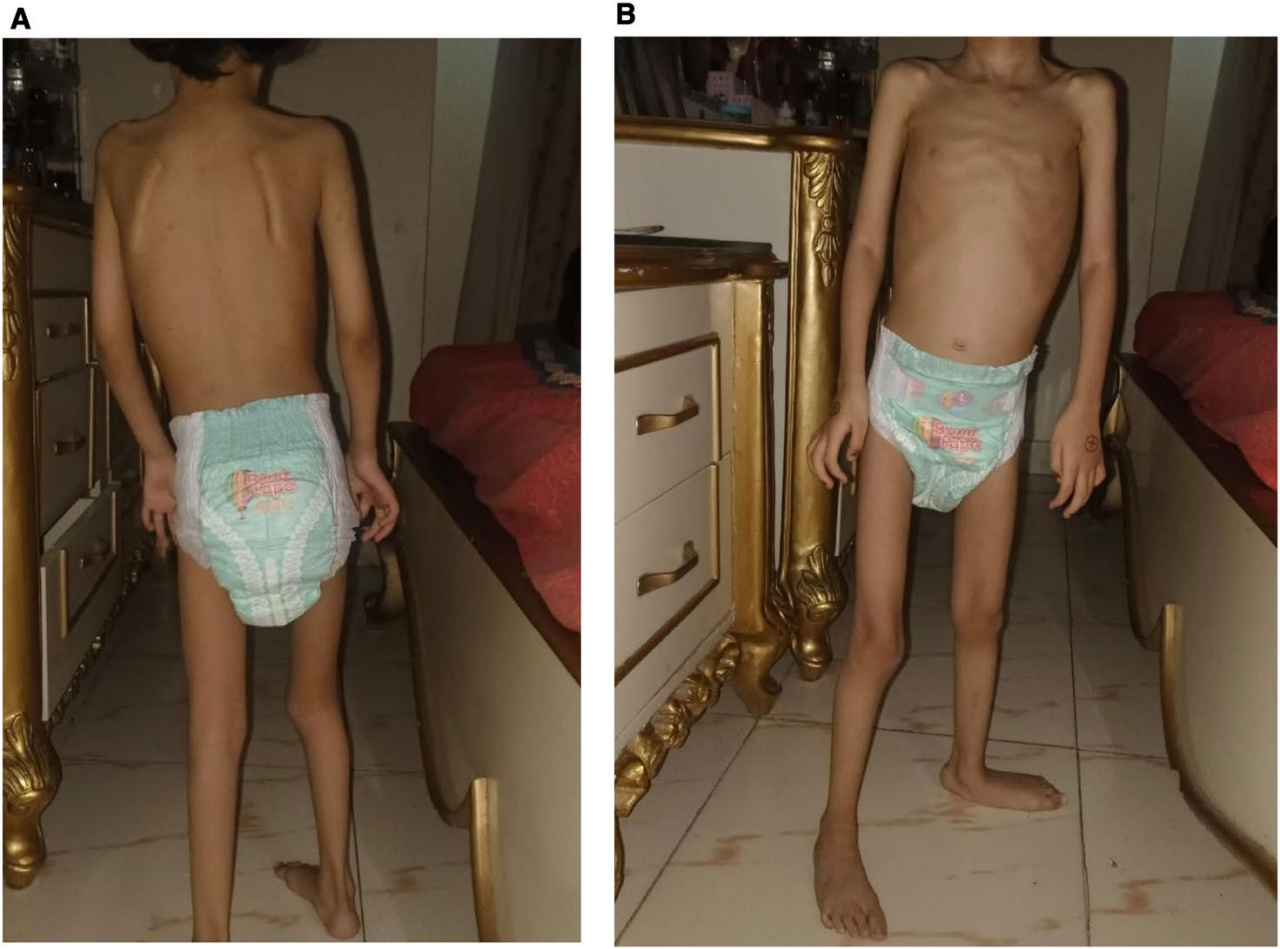
(A) and (B): Photographs of the patient (Provided by mother).

### 2.3 Timeline

**Table:** 

Timeline	Event
Birth	Preterm C‐section, severe RDS, NICU admission
0–2 weeks	Left lung collapse →Bubble CPAP 5 × 5; IV antibiotics + inotropic support
3 months	Persistent limb weakness → referral to orthopedics
4 months	DDH diagnosed; plaster cast applied
8 months	No improvement in hip; open reduction suggested but declined
12 months	Referral to neurology → physiotherapy started
2 years	Genetic testing supported autosomal recessive UCMD; both parents are carriers

### 2.4 Diagnostic Assessment

The first diagnosis of respiratory distress syndrome (RDS) was established upon delivery and corroborated through X‐ray imaging. Due to the absence of a NICU at the initial hospital, the infant was transferred to an alternative facility for treatment. The child received antibiotics and inotropic assistance. A pelvic X‐ray conducted 3 months later confirmed DDH via an orthopedic assessment (Figure 2). Following the ineffectiveness of closed reduction and cast treatment, and because of the child's significant hypotonia and failure to achieve any motor milestones, a referral to pediatric neurology was made. A clinical suspicion of congenital muscular dystrophy prompted genetic testing. The findings indicated the presence of a homozygous likely pathogenic splice‐acceptor variant in COL6A1 (c.1576‐2_1576‐1del), supporting the diagnosis of autosomal recessive UCMD when interpreted with the clinical phenotype (Table 1). Both parents were identified as carriers. No brain imaging, muscle biopsy, or electromyography was conducted due to unavailability and prohibitive costs.

**FIGURE 2:**
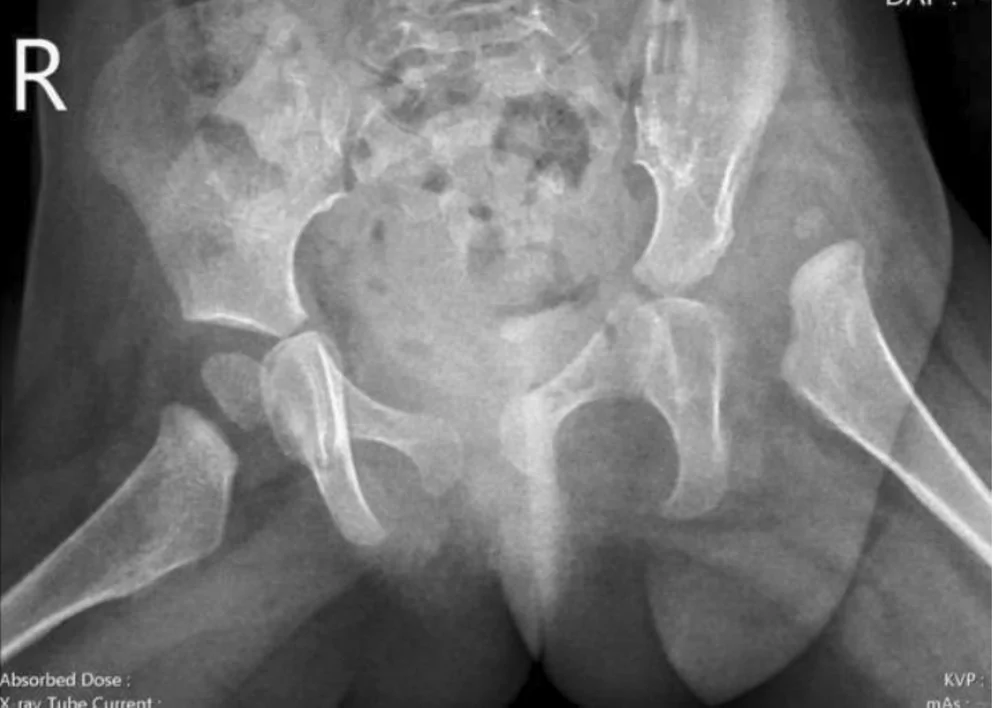
X‐ray of DDH.

**TABLE 1:** Genetic testing summary. Molecular findings and clinical interpretation of the identified COL6A1 variant.

Category	Parameter	Result
Gene/Variant	Gene/OMIM	COL6A1/120,220
Gene/Variant	Genomic coordinate (GRCh38)	chr21:45998395 CAG>C
Gene/Variant	Transcript ID	NM_001848.3
Gene/Variant	HGVS nomenclature	c.1576‐2_1576‐1del
Gene/Variant	Protein change	p.?
Variant Classification	Location	23/34
Variant Classification	Zygosity	Homozygous
Variant Classification	Function	splice_acceptor_variant
Variant Classification	Impact	HIGH
Clinical Interpretation	ClinVar	Likely pathogenic
Allele Frequency	Local Database	—
Allele Frequency	gnomAD	—
In Silico Predictors	MetaSVM	—
In Silico Predictors	SIFT	—
In Silico Predictors	Mutation Assessor	—
In Silico Predictors	MutationTaster	—
Clinical Interpretation	Clinical significance	Likely pathogenic
Clinical Interpretation	ACMG criteria	PVS1, PM2_SUP

*Note:* ACMG classification key: Class 1, pathogenic; Class 2, likely pathogenic; Class 3, variant of uncertain significance (VUS); Class 4, likely benign; Class 5, benign.Molecular analysis identified a homozygous splice‐acceptor variant in the COL6A1 gene (OMIM 120220; Transcript NM_001848.3; Genomic coordinate GRCh38: chr21:45998395 CAG〉C; HGVS nomenclature c.1576‐2_1576‐1del) located at exon 23/34. The variant is classified as a high‐impact splice acceptor variant with homozygous zygosity. According to ACMG criteria (PVS1, PM2_SUP), the variant is classified as Likely Pathogenic. This variant was absent in gnomAD and local databases.

Abbreviations: ACMG, American College of Medical Genetics and Genomics; gnomAD, Genome Aggregation Database; HGVS, Human Genome Variation Society; PM2_SUP, supporting evidence related to population frequency; PVS1, pathogenic very strong.

### 2.5 Therapeutic Interventions

The patient received inotropic support, oxygen via bubble CPAP for a collapsed left lung while in the NICU. She lacked a ventilator. She was discharged with IV antibiotics and home therapy after achieving stability. Orthopedic interventions for DDH included closed reduction and hip casting; however, these methods proved ineffective. Open reduction was suggested; however, the family declined the procedure. She was referred to physiotherapy, which provided some assistance in her functionality, enabling her to hold her neck, sit with support, and take a few steps with assistance. No drug‐specific or disease‐specific treatment was offered. Genetic counseling was accessible; nevertheless, its utilization was limited.

### 2.6 Follow‐Up and Outcomes

At the age of two, the youngster remained physically bound and was unable to move independently. She was able to sit up and walk with assistance. Cognition, social interaction, and fine motor abilities were all developmentally appropriate for her age. However, she was unable to regulate her bladder. Recurring infections of the ocular and respiratory systems raised concerns over the efficacy of the immune system. The family ceased attending physiotherapy as the patient became irritable after sessions and the parents were unaware of the importance of physiotherapy. A younger sibling was born with a similar type of hypotonia; however, genetic testing is required for confirmation. The family expressed their desire to forgo any further genetic counseling or testing for subsequent pregnancies.

## 3 Discussion

Ullrich congenital muscular dystrophy (UCMD) is a rare myopathy linked to collagen VI, originally found in 1930 [8]. The condition is marked by early‐onset hypotonia, progressive muscle weakness, hyperlaxity in distal joints, contractures in proximal joints, and exacerbating respiratory issues [9]. Alterations in the COL6A1, COL6A2, or COL6A3 genes disrupt the extracellular matrix, mostly impacting skeletal muscle [10]. UCMD is classified into two categories: autosomal dominant and autosomal recessive. The latter is more severe and is frequently associated with consanguinity [11]. This example illustrates the challenges associated with diagnosing and treating uncommon neuromuscular illnesses in resource‐limited tertiary environments. The case pertains to a premature neonate with autosomal recessive UCMD from a consanguineous family in Pakistan, who furthermore had neonatal respiratory distress, orthopedic abnormalities, a delayed diagnosis, and restricted access to NICU facilities.

In the present case, the molecular result was reported as a homozygous COL6A1 splice‐acceptor variant, c.1576‐2_1576‐1del (NM_001848.3), classified as likely pathogenic rather than definitively pathogenic. We considered this result sufficient to support the diagnosis when interpreted in the clinical context because it affects the canonical splice‐acceptor site and was assigned PVS1 with PM2_supporting in the laboratory report, indicating a predicted loss‐of‐function effect in a gene in which such variants are a recognized disease mechanism, with rarity or absence in population databases. This genetic finding was also concordant with the consanguineous family history, carrier status of both parents, and the patient's phenotype, including congenital hypotonia, severe limb weakness, distal joint laxity/contractures, orthopedic deformity, and early respiratory compromise. Muscle biopsy and collagen VI immunohistochemistry were not performed because these investigations were unavailable and financially prohibitive. Therefore, the diagnosis was based on the combination of the characteristic clinical phenotype, autosomal recessive family context, and the likely pathogenic homozygous COL6A1 variant, while acknowledging that muscle biopsy could have provided additional functional confirmation [12].

This study represents one of the initial comprehensive investigations from Pakistan addressing the natural history, challenges, and confirmed genetic diagnosis of autosomal recessive UCMD in neonates. Limited descriptions of congenital muscular dystrophies exist from South Asia, although few have examined the interplay between consanguinity, neonatal respiratory complications, and healthcare system obstacles [13, 14]. Our case is distinct as the initial indication was baby RDS, which is rarely seen in UCMD. It is widely recognized that respiratory complications arise later in the progression of UCMD, primarily due to the gradual weakening of the diaphragm. Nonetheless, the occurrence of RDS during the neonatal period is uncommon and may be readily misidentified as prematurity or sepsis, as illustrated in our case [15].

Prior studies indicate that UCMD is frequently misdiagnosed as cerebral palsy, spinal muscular atrophy, or idiopathic hypotonia, particularly in resource‐constrained settings [16]. Bönnemann et al. assert that a conclusive diagnosis is generally postponed until late infancy or childhood due to insufficient knowledge and the nonspecific nature of early indications [17]. In our case, the child did not undergo genetic testing until after several months, although exhibiting severe hypotonia and markedly delayed motor development. This occurred because orthopedic interventions were ineffective, and physiotherapy yielded only little enhancement. The postponement in diagnosis parallels the situation in Turkey and Iran, where cultural acceptance of consanguineous marriages and insufficient genetic services resulted in delayed or absent diagnoses of UCMD and other autosomal recessive disorders [18, 19].

The early orthopedic intervention for our patient with DDH illustrates the detrimental consequences of addressing structural issues without considering the underlying neuromuscular etiology. The initial closed reduction was unsuccessful, and open reduction was not feasible due to hypotonia and the associated anesthetic risks. This reflects a broader tendency in low‐ and middle‐income countries (LMICs), where muscular issues remain an overlooked factor in surgical decision‐making, particularly in pediatric orthopedics. The orthopedic failure, along with the parents' reluctance to proceed with high‐risk surgery, resulted in a neurology referral followed by genetic testing [20]. This may have occurred earlier if indicators such as joint hyperlaxity, delayed motor milestones, and a history of respiratory issues had been observed concurrently.

This example is significant as it underscores consanguinity as a primary risk factor for autosomal recessive neuromuscular disorders, particularly in South Asia, where cousin marriages are prevalent. Research from Pakistan and the Middle East indicates that between 40% and 60% of individuals are genetically connected, hence increasing the prevalence of inherited metabolic and muscular disorders [21] Genetic counseling remains underutilized, and there is a paucity of public health efforts aimed at increasing knowledge [22]. In our situation, both parents were confirmed carriers; nevertheless, prenatal screening was not conducted in the subsequent pregnancy. The younger sister has exhibited indications of hypotonia. This missed opportunity underscores the necessity of accessible and inexpensive genetic counseling services within primary maternity and child health programs.

The resource‐constrained tertiary care environment is another significant aspect of this paper. The infant was ultimately transferred to a children's hospital equipped with a NICU and specialized services; nevertheless, her initial delivery occurred at a facility without neonatal expertise and emergency resuscitation capabilities. This fragmented prenatal treatment is characteristic of LMICs due to inadequate referral systems and a shortage of pediatric neuromuscular specialists [23]. Her condition deteriorated significantly due to the absence of ventilatory support, comprehensive immunological assessment, and rehabilitative therapy. Due to lack of awareness, physiotherapy was discontinued, and long‐term orthopedic and neurological care was restricted. This illustrates the correlation between rare diseases and systemic healthcare disparities. This subject is hardly addressed in UCMD literature, predominantly derived from high‐income countries.

Contrary to previous assertions, our case demonstrates that cognitive development can remain unaffected in UCMD. For instance, our patient demonstrated the ability to acquire language, engage with people, and assimilate new information. This corroborates the findings of Bernardi et al.: despite severely impaired gross motor ability, cognitive function typically remains intact. This indicates that individuals can attain a high quality of life with appropriate assistance [23].

This situation presents several significant concerns. If hypotonia is detected early in a neonate, a comprehensive neuromuscular evaluation should be conducted, particularly in consanguineous families. Secondly, orthopedic interventions for DDH or limb deformities in hypotonic infants should be coordinated with neurologists to prevent unnecessary surgical procedures. Third, enhancing genetic diagnostic pathways and integrating them with perinatal care may facilitate earlier diagnosis and counseling, hence reducing recurrence risk and streamlining the organization of supportive treatment. Finally, governments must incorporate rare genetic disorders into their national child health strategies and ensure that tertiary care facilities are equipped with appropriate resources, including infrastructure and multidisciplinary teams comprising pediatric neurologists, geneticists, and physiotherapists.

## Author Contributions

**Kanza Farhan:** conceptualization, writing – original draft, writing – review and editing. **Muhammad Burhan Tariq:** conceptualization, writing – original draft, writing – review and editing. **Amna Farhan:** conceptualization, writing – original draft, writing – review and editing. **Erma Khalid:** supervision. **Suhaira Hafeez Syed:** writing – original draft, writing – review and editing. **Mohammed Hammad Jaber Amin:** writing – original draft, writing – review and editing.

## Funding

The authors have nothing to report.

## Disclosure

Patient perspective: The mother expressed her profound distress at the initial erroneous diagnoses and the varying physician opinions. Initially, she was skeptical and struggled both emotionally and financially with a fractured care system. Despite her limited finances, she remains actively engaged in her child's care and seeks to further her understanding of her daughter's health.

## Ethics Statement

The patient's legal guardian provided written consent for the publication of this case report and the accompanying images.

## Consent

Written informed consent from the patient was obtained according to journal guidelines.

## Conflicts of Interest

The authors declare no conflicts of interest.

## Data Availability

The data that support the findings of this study are available on request from the corresponding author. The data are not publicly available due to privacy or ethical restrictions.
